# Predictive Analysis of Brain‐Derived Neurotrophic Factor and Apolipoprotein E SNPs in Alzheimer’s Pathogenesis

**DOI:** 10.1155/bmri/3806517

**Published:** 2026-05-22

**Authors:** Muralidhara Nitheesh Beliraya, Sandeep Mallya, Sudharshan Prabhu

**Affiliations:** ^1^ Department of Bioinformatics, Manipal School of Life Sciences, Manipal Academy of Higher Education, Manipal, India, manipal.edu; ^2^ Department of Cellular and Molecular Biology, Manipal School of Life Sciences, Manipal Academy of Higher Education, Manipal, India, manipal.edu

**Keywords:** Alzheimer’s disease, APOE, BDNF, computational biology, nsSNPs single nucleotide polymorphisms, pathogenicity prediction, protein stability

## Abstract

**Background:**

Brain‐derived neurotrophic factor (BDNF) and apolipoprotein E (APOE) are key regulators of neuronal function and cognitive health. Genetic variations in these genes, particularly nonsynonymous single‐nucleotide polymorphisms (nsSNPs), have been linked to Alzheimer’s disease (AD). This study employs a computational approach to predict the potential functional impacts of nsSNPs in BDNF and APOE to explore their contributions to AD pathogenesis.

**Methods:**

A total of 3590 BDNF and 27,830 APOE SNPs were retrieved from the dbSNP database. Following quality filtering of coding region localization and minor allele frequency (≥ 0.001), 33 BDNF nsSNPs and 95 APOE nsSNPs underwent systematic analysis. Pathogenicity was assessed using SIFT and PolyPhen‐2 algorithms, with functional impact evaluated via CADD scoring. Protein stability effects were predicted using MUpro and I‐Mutant tools, and posttranslational modifications were analyzed via a GPS prediction system. Secondary structure alterations were assessed using GOR4, and three‐dimensional structural models were generated through SWISS‐MODEL with Ramachandran plot validation.

**Results:**

Three variants demonstrated concordant pathogenic predictions: rs1048218 (BDNF Q75H), rs7412 (APOE R176C), and rs769455 (APOE R163C). Protein stability analysis of these variants revealed consistent destabilization for rs1048218 (*ΔΔ*G: −1.001 to −2.08 kcal/mol) and rs7412 (*ΔΔ*G: −0.859 to −0.07 kcal/mol), whereas rs769455 showed conflicting predictions between algorithms. Posttranslational modification sites remained conserved across all the variants. Secondary structure analysis demonstrated minimal *α*‐helix reduction (0.31%–0.81%) with compensatory random coil increases. Three‐dimensional modeling revealed preserved overall protein folds despite localized structural perturbations, with acceptable model quality metrics (MolProbity scores ≤ 1.39, Ramachandran favored regions >91%).

**Conclusion:**

In silico analysis suggested that certain nsSNPs in BDNF and APOE may negatively affect protein function and stability, despite preserved structural and posttranslational features. These computational predictions need further experimental validation to better understand their roles in AD pathogenesis.

## 1. Introduction

Alzheimer’s disease (AD) is a neurodegenerative disorder characterized by damage to nerve cells in the brain. It is the most common type of dementia, primarily affecting individuals aged 60 and above [1]. The key pathological hallmarks of AD include the accumulation of amyloid‐*β* (A*β*) plaques and tau‐containing neurofibrillary tangles (NFTs) [2]. These changes lead to memory loss, neuronal dysfunction, cognitive decline, impaired verbal communication, neuroinflammation, and chronic neuronal loss [3]. On the basis of symptoms, the progression of AD is typically categorized into three stages: early, intermediate, and late [4, 5].

Genetic factors significantly contribute to AD development, with brain‐derived neurotrophic factor (BDNF) and apolipoprotein E (APOE) being considered two of the major genetic determinants [6]. Although the precise mechanism underlying AD pathogenesis remains unclear, early‐onset AD is often associated with mutations in the genes encoding amyloid precursor protein (APP), presenilin 1 (PSEN1), and presenilin 2 (PSEN2) [7]. In contrast, APOE is a major genetic contributor to late‐onset AD, accounting for approximately 95% of cases. The human APOE gene exists in three alleles, *ε*2 (APOE2), *ε*3 (APOE3), and *ε*4 (APOE4), with the *ε*4 allele identified as the strongest genetic risk factor for AD [8].

BDNF is a neurotrophin that supports neuronal survival, differentiation, morphology, development and synaptic remodeling. Human BDNF is located on chromosome 11p14.1 [9]. Studies suggest that reduced levels of BDNF are associated with A*β* accumulation, neuroinflammation, neuronal cell death, and tau phosphorylation. However, the exact mechanism by which BDNF is disrupted in AD remains unclear [10]. APOE, on the other hand, is synthesized primarily by the liver and macrophages in peripheral tissues, where it plays a critical role in lipid transport and homeostasis [11]. In the central nervous system (CNS), astrocytes and microglia are the main sources of APOE. During synaptic plasticity and membrane repair, APOE facilitates the delivery of cholesterol and lipids to neurons through APOE receptors. Even a single amino acid substitution can alter the binding affinity of APOE isoforms to receptors, lipids, and A*β*, influencing A*β* accumulation and distribution [12].

Single‐nucleotide polymorphisms (SNPs) are the most common genetic variations in humans and are often implicated in a range of complex diseases. Among them, nonsynonymous single‐nucleotide polymorphisms (nsSNPs) are critical, as they result in amino acid substitutions that can alter protein function and structure [13]. These changes may affect enzyme activity, disrupt transcription factor binding and ultimately impair gene expression, contributing to various genetic disorders [1].

Despite increasing genetic evidence, the specific functional consequences of nsSNPs in BDNF and APOE remain incompletely understood. To bridge this knowledge gap, in silico approaches provide a rapid and cost‐effective method to predict the pathogenic potential and structural impact of these variants. This study employs a range of computational tools to analyze nsSNPs in the human BDNF and APOE genes and evaluate their potential influence on protein function and stability.

## 2. Methods

### 2.1. Ethical Considerations

This study exclusively employed computational approaches and did not involve human participants, animal models, or laboratory experimentation. Therefore, ethical approval and informed consent were not applicable to this study.

### 2.2. Sampling Approach and Justification

**Figure 1:**
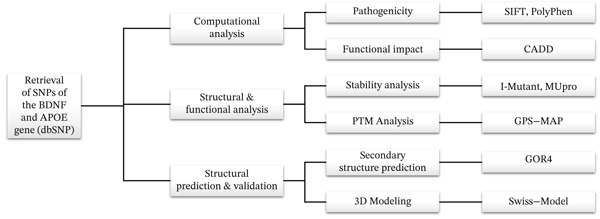
Workflow for SNP retrieval, inclusion, and analysis using in silico approaches.

The overall workflow of this study is outlined in Figure 1. The SNP data for the BDNF and APOE genes were retrieved from dbSNP via a targeted search approach [14]. This approach allowed us to focus on genes with well‐established associations with AD.

### 2.3. Inclusion Criteria

• SNPs located in the coding region of BDNF and APOE
• Nonsynonymous SNPs with minor allele frequency (MAF) ≥ 0.001

### 2.4. Exclusion Criteria

• Intronic and synonymous SNPs
• SNPs with an MAF < 0.001

### 2.5. SNP Retrieval and Dataset Compilation

Database queries were performed to identify nonsynonymous SNPs from both target genes. Following systematic filtering and application of the inclusion and exclusion criteria, a refined dataset was obtained for comprehensive analysis. Each SNP entry included the SNP ID, chromosomal location, nucleotide substitution, and resulting amino acid change. Subsequent analyses focused exclusively on common nsSNPs predicted as pathogenic by both the SIFT and PolyPhen‐2 algorithms to ensure analytical stringency.

### 2.6. Computational Prediction Analyses

#### 2.6.1. Functional Impact Prediction

The Combined Annotation Dependent Depletion (CADD) framework was employed to evaluate the functional consequences of the identified nsSNPs [15]. PHRED‐scaled scores were interpreted according to established guidelines: scores ≥ 10 represent the 10% most deleterious possible substitutions, scores ≥ 20 indicate the 1% most deleterious, and scores ≥ 30 represent the 0.1% most deleterious variants genome‐wide. We considered all variants above a score of 20 for further analysis.

#### 2.6.2. Pathogenicity Prediction

SIFT Algorithm. The Sorting Intolerant From Tolerant (SIFT) tool was utilized to predict variant deleteriousness [16]. Variants exhibiting SIFT scores < 0.05 were classified as deleterious, whereas those with scores ≥ 0.05 were considered tolerated.

PolyPhen‐2 Analysis. Polymorphism Phenotyping v2 serves as a complementary pathogenicity prediction tool [17]. The scoring continuum (0.00–1.00) was interpreted as follows: 0.00–0.15 (benign), 0.15–0.85 (possibly damaging), and 0.85–1.00 (probably damaging).

#### 2.6.3. Consensus Variant Selection

Only variants demonstrating concordant predictions (deleterious by SIFT and likely/probably damaging by PolyPhen‐2) were selected for subsequent in silico characterization, ensuring high confidence in the pathogenicity predictions.

### 2.7. Structural and Functional Analysis

#### 2.7.1. Protein Stability Analysis

Thermodynamic stability changes resulting from amino acid substitutions were evaluated using two complementary sequence‐based prediction tools, namely, MUpro and I‐Mutant [18, 19]. Both of these tools calculate Gibbs free energy changes (*ΔΔ*G), where negative values indicate decreased stability and positive values suggest increased stability relative to that of wild‐type proteins.

#### 2.7.2. Posttranslational Modification Analysis

Group‐based prediction system (GPS) tool was used to identify potential alterations in phosphorylation patterns [20]. Both the wild‐type and variant protein sequences of BDNF and APOE were analyzed under the CK1 kinase model to detect possible modifications in terms of phosphorylation site availability and kinase specificity.

#### 2.7.3. Secondary Structure Prediction

The Garnier–Osguthorpe–Robson version 4 (GOR4) algorithm was utilized to predict secondary structure elements, categorizing amino acid residues into *α*‐helices, *β*‐strands, and random coils on the basis of primary sequence information [21]. Comparative analysis between the wild‐type and variant sequences was performed to detect mutation‐induced alterations in secondary structure composition.

### 2.8. Three‐Dimensional Structural Modeling and Validation

Three‐dimensional protein structures for identified variants were generated using the SWISS‐MODEL homology modeling platform [22]. The models with the highest global model quality estimation (GMQE) score were selected to ensure reliability. Structural validation was performed through Ramachandran plot analysis to assess the backbone dihedral angle distributions and overall stereochemical quality.

All the generated mutant models demonstrated acceptable structural quality on the basis of comprehensive MolProbity assessments, including favorable overall MolProbity scores and minimal steric clash scores, thereby supporting the reliability of the subsequent structural predictions and analyses.

## 3. Results

### 3.1. Functional Impact Evaluation via CADD Scoring

The CADD analysis identified high‐impact variants with PHRED scores ≥ 20. Ten BDNF variants presented scores ranging from 20.4 to 31.0, whereas 12 APOE variants presented scores between 23.3 and 56.0, indicating a substantial likelihood of deleterious functional consequences (Table 1).

**Table 1:** High‐impact variants with CADD PHRED scores ≥ 20.

Gene	rsIDs	Ref	Alt	Raw score	PHRED
BDNF	rs1049779568	T	G	5.3057	29.8
	rs1590216799	T	G	5.3686	31
	rs1048218	C	A	3.3057	22.1
	rs139352447	G	C	2.8056	20.4
	rs6265	C	A	3.1638	21.6
	rs6265	C	G	3.1699	21.6
	rs6265	C	T	4.1758	24.5
	rs8192466	G	A	3.8198	23.6
	rs8192466	G	T	3.3397	22.2
	rs8192466	G	C	2.9853	21
APOE	rs752693941	G	T	12.0308	56
	rs769455	C	T	5.1062	28.6
	rs121918393	C	T	4.9265	27.5
	rs121918393	C	A	4.2803	24.8
	rs7412	C	T	4.3721	25.1
	rs868094551	C	A	3.9388	23.9
	rs767339630	G	A	3.924	23.8
	rs199768005	T	A	3.9103	23.8
	rs573658040	C	T	3.6962	23.3
	rs1599954391	T	G	3.8682	23.7
	rs140808909	G	T	7.9369	36
	rs557715042	G	A	8.7425	38

### 3.2. Identification and Pathogenicity Prediction of Nonsynonymous SNPs

Comprehensive database mining of dbSNP retrieved 3590 SNPs from the BDNF gene and 27,830 SNPs from the APOE gene. Initial filtering identified 505 nonsynonymous SNPs (nsSNPs) in BDNF and 375 nsSNPs in APOE. The application of stringent inclusion criteria (coding region localization, minor allele frequency ≥ 0.001, and nonsynonymous variants) yielded a refined dataset of 33 BDNF nsSNPs and 95 APOE nsSNPs for comprehensive computational analysis.

### 3.3. Consensus Pathogenicity Predictions

SIFT analysis predicted 4 nsSNPs in BDNF and 17 in APOE as deleterious (score < 0.05), whereas PolyPhen‐2 classified 3 BDNF variants and 4 APOE variants as probably damaging (score ≥ 0.85). Critically, three variants demonstrated concordant predictions across both algorithms: rs1048218 in BDNF and rs7412 and rs769455 in APOE (Table 2). The BDNF variant rs1048218 (Q75H) exhibited moderate deleteriousness (SIFT: 0.041) with a high damage probability (PolyPhen‐2: 0.956). Both APOE variants presented maximal PolyPhen‐2 scores (1.000) and very low SIFT scores, indicating that strong pathogenic potential affects critical arginine residues involved in lipid binding.

**Table 2:** Consensus pathogenicity predictions for high‐confidence variants.

Gene	SNP ID	Amino acid change	SIFT score	SIFT prediction	PolyPhen‐2 score	PolyPhen‐2 prediction
BDNF	rs1048218	Q75H	0.041	Deleterious	0.956	Probably damaging
APOE	rs7412	R176C	0.001	Deleterious	1.000	Probably damaging
APOE	rs769455	R163C	0.0080	Deleterious	1.000	Probably damaging

To contextualize the functional relevance of the identified variants within established AD biomarker frameworks, the predicted effects of rs1048218 (BDNF Q75H), rs7412 (APOE R176C), and rs769455 (APOE R163C) were interpreted against core AD pathological hallmarks. Reduced BDNF levels in the cerebrospinal fluid and hippocampus are a well‐replicated feature of AD, correlating with A*β* plaque burden, tau phosphorylation, and episodic memory decline core diagnostic criteria per the NIA‐AA research framework [23]. The predicted destabilization of BDNF Q75H (*ΔΔ*G: −1.001 to −2.08 kcal/mol) provides a plausible molecular basis for impaired mature BDNF availability, potentially accelerating A*β* accumulation and neurofibrillary tangle formation [10, 24]. For APOE variants, rs7412 defines the *ε*2 allele, which is associated with reduced A*β* plaque deposition in post‐mortem AD brains [8, 25]; rs769455 has been identified as a population‐specific risk modifier in APOE haplotype analyses. While these variants were retrieved from general population databases, their inclusion was governed by functional criteria directly relevant to AD, neurotrophin signaling integrity and apolipoprotein‐mediated lipid and A*β* homeostasis.

### 3.4. Protein Stability Analysis

Thermodynamic stability assessment using MUpro and I‐Mutant 2.0 revealed predominantly destabilizing effects for the consensus variants (Table 3). Both tools concordantly indicated substantial destabilization for rs1048218 (BDNF, Q75H), with I‐Mutant predicting a more pronounced effect (*ΔΔ*G = ‐2.08 kcal/mol). For APOE rs7412 (R176C), both predictors suggested destabilization, although the effect was modest in the I‐Mutant group (*ΔΔ*G = ‐0.07 kcal/mol), indicating a likely mild reduction in protein stability. In contrast, APOE rs769455 (R163C) yielded conflicting predictions, suggesting marginal structural perturbations. Taken together, these findings suggest strong destabilization for BDNF Q75H, moderate destabilization for APOE R176C, and an overall neutral to marginal effect for APOE R163C.

**Table 3:** Protein stability predictions for consensus variants.

Gene	RSID	Mutation	Tool	*ΔΔ*G (kcal/mol)	Prediction (per tool)	Consensus interpretation
BDNF	rs1048218	Q75H	MUpro	−1.001	Decrease stability	Destabilizing (strong evidence, both tools agree)
			I‐Mutant 2.0	−2.08	Decrease stability	

APOE	rs7412	R176C	MUpro	−0.859	decrease stability	Likely destabilizing (moderate evidence, mild effect in I‐Mutant)
			I‐Mutant 2.0	−0.07	Decrease stability	
	rs769455	R163C	MUpro	−0.76	Decrease stability	Uncertain/likely neutral (predictions conflict, both near zero)
			I‐Mutant 2.0	0.08	Increase stability	

### 3.5. Posttranslational Modification Analysis

Phosphorylation site predictions via GPS‐MSP demonstrated remarkable conservation of regulatory sites across wild‐type and variant sequences (Table 4). All identified phosphorylation sites remained functionally intact across variants, with prediction scores consistently exceeding threshold cutoffs. This conservation suggests minimal disruption of posttranslational regulatory mechanisms.

**Table 4:** Phosphorylation site conservation analysis.

Gene	Sequence type	Position	Residue	Kinase	Score	Status
BDNF	Wild‐type/rs1048218	39	T	CK1	0.203	Conserved
		123	S	CK1	0.183	Conserved
		130	S	AGC/CK1	0.135/0.176	Conserved
		145	S	CK1	0.116	Conserved
APOE	Wild‐type/variants	40	S	CK1	0.156	Conserved

### 3.6. Secondary Structure Impact Assessment

Secondary structure analysis via GOR4 revealed minimal but consistent structural perturbations (Table 5). All the variants presented minimal *α*‐helix reduction with compensatory increases in random coil content. Critically, no novel secondary structure elements emerged, suggesting preservation of the fundamental protein architecture.

**Table 5:** Results of secondary structure prediction of the wild type and nsSNPs of the BDNF and APOE genes.

Gene	Structure element	Wild‐type	Variant	Change (%)
BDNF (rs1048218)	*α*‐Helix	50 (20.24%)	48 (19.43%)	−0.81
	Extended strand	73 (29.55%)	73 (29.55%)	0
	Random coil	124 (50.20%)	126 (51.01%)	0.81
APOE (rs7412)	*α*‐Helix	259 (81.70%)	257 (81.07%)	−0.63
	Extended strand	9 (2.84%)	9 (2.84%)	0
	Random coil	49 (15.46%)	51 (16.09%)	0.63
APOE (rs769455)	*α*‐Helix	259 (81.70%)	258 (81.39%)	−0.31
	Extended strand	9 (2.84%)	9 (2.84%)	0
	Random coil	49 (15.46%)	50 (15.77%)	0.31

**Figure 2:**
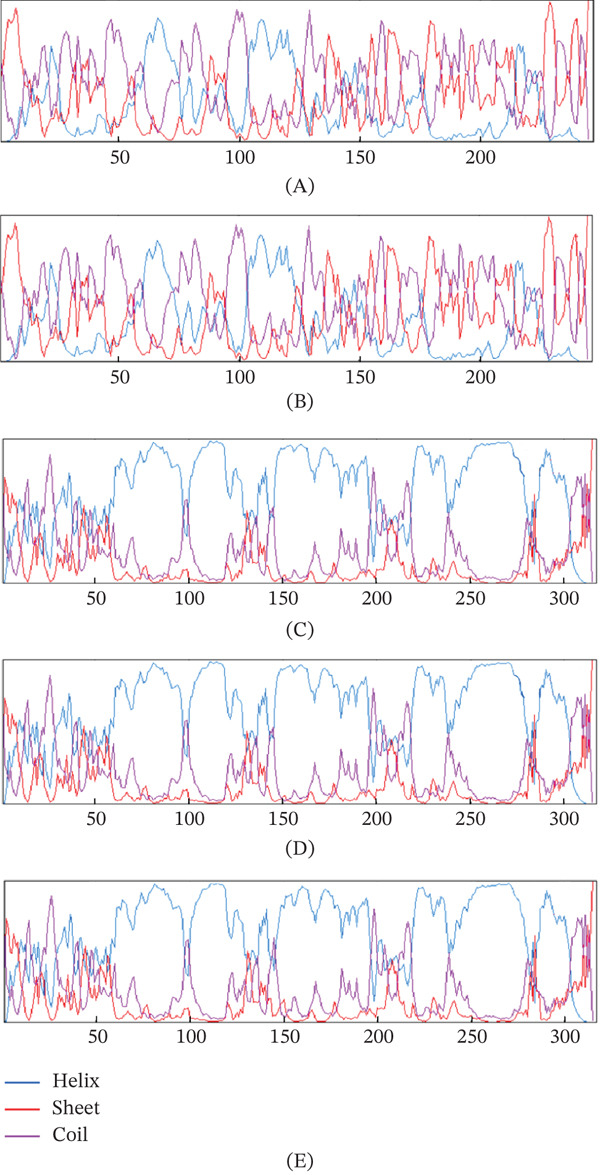
Secondary structure prediction profiles generated by the GOR4 algorithm for wild‐type and variant proteins. The plots display secondary structure probability distributions across the protein sequence (the *x*‐axis represents the amino acid position), with colored lines indicating different structural elements: red (*α*‐helix), blue (*β*‐sheet), and magenta (random coil). (A) Wild‐type BDNF protein showing a characteristic neurotrophin secondary structure distribution with balanced *α*‐helical and *β*‐sheet regions interspersed with flexible coil regions. (B) BDNF rs1048218 variant demonstrating minimal perturbations in secondary structure propensities, with subtle shifts in the *α*‐helix and compensatory increases in random coil regions while maintaining overall structural architecture. (C) Wild‐type APOE protein exhibiting the characteristic apolipoprotein secondary structure profile dominated by *α*‐helical content with minimal *β*‐sheet structure. (D) APOE rs7412 variant showing conserved secondary structure elements with minor *α*‐helix reduction and slight random coil increase. (E) The APOE rs769455 variant displays similar conservation patterns, with minimal *α*‐helix reduction and modest random coil increase.

Visual analysis of secondary structure distributions (Figure 2) corroborated these quantitative findings. Wild‐type BDNF (Figure 2A) displayed characteristic *α*‐helical (red), *β*‐sheet (blue), and coil (magenta) distributions indicative of stable neurotrophin architecture. The rs1048218 variant (Figure 2B) presented localized perturbations in the helix and coil regions while maintaining overall structural integrity. Similarly, wild‐type APOE (Figure 2C) exhibited the expected predominant *α*‐helical conformation typical of apolipoproteins. Variants rs7412 (Figure 2D) and rs769455 (Figure 2E) demonstrated subtle alterations in helical and coil compositions without introducing novel structural elements, confirming preservation of the fundamental apolipoprotein fold.

**Figure 3:**
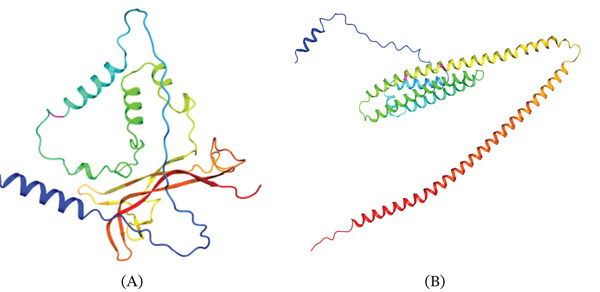
Homology‐based three‐dimensional structural models of pathogenic variants in the BDNF and APOE proteins. The proteins are represented as ribbon diagrams in the rainbow (blue to red) scheme from the N‐terminal to the C‐terminal. (A) BDNF mutant structure with *α*‐helices and *β*‐strands and the mutation site colored magenta, suggesting possible local folding changes. (B) APOE mutant structure with the mutation sites shown in magenta within *α*‐helical‐rich domains, suggesting a potential impact on folding, spatial organization, and overall protein stability and function.

**Figure 4:**
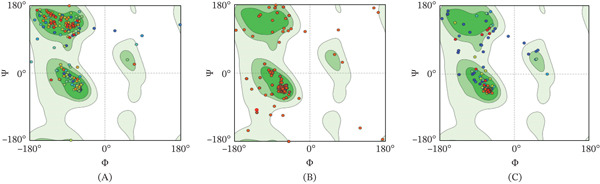
Ramachandran plot validation of three‐dimensional structural models for BDNF and APOE variants. Plots display phi (*φ*) and psi (*ψ*) backbone dihedral angles for all amino acid residues, with contoured regions indicating sterically allowed conformations. (A) The rs1048218 variant of the BDNF gene shows residues falling within favored and allowed regions with a slightly higher percentage of outliers. (B) The rs7412 variant of the APOE gene shows a high percentage of residues in the favored and allowed regions. Variants showing no significant outliers. (C) The rs769455 variant of the APOE gene shows a high percentage of residues in the favored and allowed regions. All the models meet acceptable quality thresholds for structural reliability (MolProbity scores ≤ 1.39).

All the models achieved acceptable quality metrics, with MolProbity scores ≤ 1.39 indicating reliable structural predictions. APOE variants demonstrated superior stereochemical quality with minimal Ramachandran outliers (< 1%), whereas the BDNF variant presented slightly elevated outlier percentages (4.08%), suggesting localized conformational strain. The mutation is highlighted in magenta, highlighting its spatial location within the structure (Figure 3A,B). The preserved structure suggests that these mutations are unlikely to disrupt overall protein folding. Ramachandran plot analysis further confirmed the structural reliability of the models (Figure 4B,C). The results of the Ramachandran plot analysis for the BDNF and APOE mutants are shown in Table 6.

**Table 6:** Results of structure validation via Ramachandran plots for the BDNF and APOE gene mutants.

Gene	rsID	MolProbity score	Clash score	Ramachandran favored (%)	Ramachandran outliers (%)
BDNF	rs1048218	1.36	0.00	91.02%	4.08%
APOE	rs7412	1.39	1.38	95.56%	0.63%
APOE	rs769455	1.39	1.38	95.56%	0.63%

Ramachandran plot analysis confirmed the overall structural integrity, with the majority of residues occupying the favored conformational space (Figure 4A–C). The elevated outlier percentage in BDNF rs1048218 indicates potential local backbone perturbations that may influence protein dynamics without compromising global fold stability.

## 4. Discussion

This comprehensive computational analysis identified three high‐confidence pathogenic variants‐rs1048218 (BDNF), rs7412 (APOE), and rs769455 (APOE) with potential implications for AD pathogenesis. Convergent evidence from multiple prediction algorithms provides mechanistic insights into how these variants may contribute to neurodegeneration through distinct molecular pathways.

### 4.1. Functional Implications of BDNF rs1048218

The BDNF variant rs1048218 (Q75H) demonstrated consistent destabilizing effects across stability prediction algorithms (*ΔΔ*G: −1.001 to −2.08 kcal/mol), indicating significant perturbation of the neurotrophin fold architecture. The glutamine‐to‐histidine substitution introduces a positively charged, bulkier residue that likely disrupts local hydrogen bonding networks critical for protein stability. The observed *α*‐helix reduction and compensatory random coil increase support this interpretation, suggesting that localized structural perturbations that may impair BDNF‐receptor interactions are essential for synaptic plasticity and neuronal survival.

Previous studies have reported variable associations between rs1048218 and AD risk across different populations, although evidence remains limited and population‐dependent [26]. Our computational analysis provides mechanistic support for these associations by demonstrating how this variant may compromise BDNF’s neuroprotective signaling cascade. However, the moderate CADD scores (PHRED: 31.0) suggest subtle effects that may require additional genetic or environmental factors for phenotypic manifestation, which is consistent with variable population‐specific associations. Clinically, BDNF protein levels in the cerebrospinal fluid and serum of patients with AD are significantly reduced compared with age‐matched controls, a finding replicated across multiple independent cohort studies [24]. This reduction correlates with hippocampal atrophy and episodic memory decline, consistent with the AD biomarker framework established above. Our prediction that rs1048218 destabilizes the BDNF fold (*ΔΔ*G up to −2.08 kcal/mol) provides a molecular basis for this observed reduction: a structurally compromised BDNF protein may undergo accelerated proteasomal degradation or impaired secretion via the regulated secretory pathway [26]. This is mechanistically consistent with the well‐studied Val66Met variant (rs6265), which reduces activity‐dependent BDNF secretion and is associated with hippocampal volume loss in patients with AD [27]. Although Q75H affects a distinct structural region, it may similarly impair pro‐BDNF to mature BDNF processing, warranting direct experimental comparison in future studies. The moderate CADD score (PHRED: 22.1) for rs1048218 suggests that its clinical penetrance may be modulated by polygenic background and environmental co‐factors [26]. This is consistent with the broader pattern of BDNF variant associations with AD showing population‐dependent heterogeneity, where certain SNPs show significant associations in East Asian cohorts but not in European populations [28, 29].

### 4.2. APOE Variants and Lipid Metabolism Disruption

The rs7412 variant, which defines the APOE *ε*2 allele, presents an intriguing computational paradox. Despite consistent pathogenicity predictions (SIFT: 0.001, PolyPhen‐2: 1.000), epidemiological evidence has demonstrated reduced AD risk in *ε*2 carriers compared with *ε*4 carriers [24]. This apparent contradiction may reflect complex relationships between protein stability and biological function. The arginine‐to‐cysteine substitution at position 176 occurs within the lipid‐binding domain, potentially altering lipid cargo specificity rather than eliminating function. The predicted destabilization may increase APOE turnover or modify amyloid‐*β* interactions in ways that reduce pathological aggregation, which aligns with protective epidemiological observations. The apparent discordance between the pathogenic computational prediction for rs7412 (APOE *ε*2 allele) and its well‐established epidemiological protection against AD warrants mechanistic elaboration. The R176C substitution eliminates a positively charged arginine critical for LDLR binding, which our analysis suggests is mildly destabilizing (*ΔΔ*G: −0.859 to −0.07 kcal/mol). However, this altered binding affinity may redirect APOE2 toward preferential interaction with heparan sulfate proteoglycans (HSPGs), potentially enhancing A*β* clearance through alternative internalization pathways [12]. Importantly, neuropathological studies have additionally demonstrated that APOE2 carriers exhibit reduced tau tangle burden independent of A*β* load [25], suggesting that APOE2’s protective mechanism may operate through tau‐related pathways not captured by single‐variant pathogenicity classifiers. This underscores a fundamental limitation of applying algorithms such as SIFT and PolyPhen‐2 to variants whose disease relevance is governed by isoform‐level biology and genetic context rather than isolated amino acid function.

The rs769455 variant exhibited conflicting stability predictions between algorithms, reflecting the subtle nature of R163C substitution effects. Located near the lipid‐binding domain, this variant likely modulates lipid–cargo interactions without drastically altering the overall structure. Few studies have identified rs769455 as a population‐specific risk modifier, although its low prevalence has constrained comprehensive analysis [30]. The conflicting stability predictions for rs769455 (R163C) reflect biologically meaningful uncertainty rather than mere algorithmic discordance. Residue R163 resides in the hinge region between the N‐terminal receptor‐binding domain and the C‐terminal lipid‐binding domain of APOE, a region whose inter‐domain flexibility critically governs lipid cargo exchange and receptor binding kinetics [8]. A cysteine substitution at this position introduces a free sulfhydryl group that could participate in aberrant disulfide bonding under the oxidative stress conditions prevalent in the AD brain. A mechanistic parallel may be drawn with APOE3 Christchurch (R136S), a hinge‐region variant associated with dramatically delayed AD onset in a PSEN1 mutation carrier [31]. While rs769455 and APOE3 Christchurch affect distinct positions, this comparison illustrates how subtle modifications in this inter‐domain hinge can produce disproportionate phenotypic effects that are difficult to capture using sequence‐based stability algorithms alone. Notably, rs769455 has recently been identified as an African ancestry‐specific AD risk modifier in a large case–control study of nearly 32,000 participants [32], further underscoring the importance of ancestry‐stratified analyses for this variant. The exceptional CADD scores observed for some APOE variants (PHRED: 56.0) underscore the critical importance of intact apolipoprotein function in neurological health.

### 4.3. Structural Resilience and Therapeutic Implications

The conservation of phosphorylation sites across all variants indicates that these mutations primarily affect protein structure rather than regulatory control mechanisms. This finding has important therapeutic implications, suggesting that interventions targeting posttranslational modifications may remain effective regardless of variant status. For BDNF, preserved CK1 and AGC kinase sites indicate normal upstream signaling pathway function, suggesting that therapeutic strategies enhancing BDNF expression or receptor sensitivity may compensate for structural deficits.

The maintenance of overall protein folds despite predicted destabilization suggests considerable structural resilience in both proteins. This resilience may explain why these variants exist in populations without severe developmental consequences while contributing to late‐onset disease susceptibility. The subtle secondary structure alterations (*α*‐helix to random coil transitions) may represent adaptive flexibility, allowing proteins to maintain essential functions while altering specialized activities relevant to neurodegeneration. From a translational perspective, the three variants identified in this study map onto distinct nodes of the AD pathophysiological cascade with direct therapeutic relevance. BDNF rs1048218 affects the neurotrophin survival signaling axis, which is already being targeted clinically through TrkB agonists and BDNF mimetics in early‐phase trials. APOE rs7412 and rs769455 affect lipid homeostasis and A*β* clearance pathways, which are the focus of APOE‐directed therapies including antisense oligonucleotides and small molecule APOE modulators currently under investigation [8]. Although the present study does not provide experimental validation, the mechanistic specificity of the predicted variant effects suggests that genotyping these loci may contribute to the stratification of patients for precision‐targeted interventions. Future studies integrating these computational predictions with PET A*β*/tau imaging data, CSF biomarker profiles (p‐tau181, A*β*42/40 ratio), and longitudinal cognitive outcomes from established AD cohorts such as Alzheimer’s Disease Neuroimaging Initiative (ADNI) or the Alzheimer’s Disease Sequencing Project (ADSP) would be particularly valuable to translate these findings into clinically actionable knowledge for AD prevention and treatment.

## 5. Conclusion

Through a comprehensive multitool integrative framework, we identified three high‐confidence variants of potential pathogenic relevance, namely, rs1048218 in BDNF and rs7412 and rs769455 in APOE, that may contribute to AD pathogenesis. These variants were consistently prioritized across diverse computational pipelines encompassing pathogenicity prediction, protein stability assessment, and structural modeling, underscoring their functional relevance. Specifically, they exhibited (i) concordant deleterious predictions across multiple in silico pathogenicity classifiers, (ii) thermodynamic destabilization with varying magnitudes, (iii) preservation of regulatory and posttranslational modification sites, (iv) subtle structural perturbations without fold disruption, and (v) acceptable model quality supporting prediction reliability. Collectively, these findings make the identified variants prime for functional validation studies to elucidate their roles in the neurotrophin signaling (BDNF) and lipid metabolism (APOE) pathways, which are relevant to AD.

### 5.1. Rationale of the Study

AD is influenced by multiple genetic factors, with APOE and BDNF being the most important contributors of risk. While their links to the disease are well established through epidemiological studies. The exact impact of specific genes nonsynonymous SNPs on protein function is still not clear. This study aimed to bridge the gap through an integrative computational approach to identify variants most likely to alter proteins stability and function. The exclusive focus on nonsynonymous coding variants in BDNF and APOE, our findings provide mechanistic insights and establish a basis for precision‐medicine strategies focused on genetically at‐risk groups of patients with AD.

### 5.2. Limitation of the Study

This study relied majorly on computational predictions, which cannot fully capture the complexity of biological systems. Differences between prediction algorithms highlight the uncertainty of these tools, especially for variants with borderline effects. Our analysis was limited to nonsynonymous coding SNPs, excluding regulatory and splice‐site variants that could also influence gene expression and disease risk. The absence of population‐specific analyses limits generalizability across diverse genetic backgrounds. Given the established population differences in APOE allele frequencies and effects, ancestry‐specific validation will be crucial for clinical translation. Experimental validation through protein expression studies, functional assays, and clinical correlation studies will be essential to confirm the biological relevance of these computational predictions.

Despite these limitations, the identified variants represent valuable targets for precision medicine in AD. The mechanistic insights provided could guide the development of variant‐specific therapeutic interventions, particularly for carriers of high‐impact APOE variants. Understanding the structural basis of variant effects may facilitate the design of small molecule stabilizers or functional modulators that ameliorate pathogenic consequences while preserving beneficial protein activities. Future studies integrating experimental validation with population‐specific genomic data will be crucial to translate these computational insights into clinically actionable knowledge for AD prevention and treatment strategies.

## Author Contributions

**Muralidhara Nitheesh Beliraya:** literature review, interpretation of data, original drafting of the manuscript, revision of the manuscript; **Sandeep Mallya:** data compilation, interpretation of data, original drafting of the manuscript, revision of the manuscript; **Sudharshan Prabhu:** study conception, literature review, critical revision of the manuscript. **Muralidhara Nitheesh Beliraya and Sandeep Mallya** have contributed equally to this manuscript.

## Funding

This study was supported by Indian Council of Medical Research, 10.13039/501100001411, 54/8/GER/2019‐NCD‐II; DBT‐BUILDER, BT/INF/22/SP43065/2021; Manipal Research Board Grant, MRB Grant; MAHE Seed Money Grant Seed Money.

## Disclosure

This paper has been uploaded to ResearchSquare as a preprint: https://www.research-square.com/article/rs-4423359/v1.

## Conflicts of Interest

The authors declare no conflicts of interest.

## Data Availability

The data that support the findings of this study are available from the corresponding author upon reasonable request.
